# The association between the rate of potentially pharmacist-manageable emergency department visits and community income level and primary care provider availability: A spatial analysis

**DOI:** 10.1177/17151635221087621

**Published:** 2022-03-30

**Authors:** Mhd Wasem Alsabbagh, Sherilyn K. D. Houle

**Affiliations:** School of Pharmacy and the Ontario Pharmacy Evidence Network (OPEN), University of Waterloo, Kitchener, Ontario

## Introduction

In 2019, the Ontario government announced that it will introduce a “minor ailments” (referred to in this article as “ambulatory conditions”) management program for pharmacists.^1^ Currently, the Ontario Ministry of Health and the Ontario College of Pharmacists are establishing the scope parameters for this program, including eligible conditions and therapeutic options, with remuneration also yet to be established.^2^

In previous work by our group, the rate of avoidable emergency department (ED) visits that can potentially be managed by pharmacists with the ability to treat ambulatory conditions in Ontario was estimated using administrative databases.^3^ Overall, it was found that more than one-third (34.8%) of avoidable visits could potentially be managed by a pharmacist, representing almost 4.3% of all ED visits.^3^

Pharmacists are considered to be highly accessible primary health care providers in Canada,^4,5^ and most Canadians live close to a community pharmacy.^6^ This accessibility, coupled with pharmacists practising to full scope, enables them to offer timely clinical services to patients, particularly those who may have significant barriers preventing them from accessing other health care services appropriately, such as low-income populations.^7,8^ However, research in both the United States and Canada has identified that the availability of pharmacy services may be associated with an area’s socioeconomic status.^9-12^ For example, a 2018 study identified geographic disparities in the accessibility of pharmacies in the Greater Toronto Area,^11^ and research by our group has also identified that pharmacists in Alberta with additional prescribing authorization are more concentrated in high-income areas.^13^ It is currently unknown whether similar disparities exist related to the future management of ambulatory conditions by pharmacists in Ontario. The aim of this study is to determine the spatial pattern of potentially pharmacist-manageable avoidable ED visits in Ontario and to identify whether an association exists between the rate of potentially pharmacist-manageable ED visits for ambulatory conditions and a community’s characteristics, including income level and community pharmacy and other primary care provider availability.

## Methods

### Data sources

A retrospective population-based spatial analysis of provincial administrative record-level data in Ontario was performed as part of a subanalysis of our previous work, from which details on data acquisition for ED visits in Ontario can be obtained.^3^ In brief, data on ED visits in Ontario from fiscal year 2010-11 through 2016-17 were obtained from the National Ambulatory Care Reporting System database.^14^ To be considered in our analysis, an ED visit had to meet the following inclusion criteria: 1) be unscheduled; 2) result in a discharge home without admission; 3) be assigned a Canadian Triage Acuity Scale value of IV or V (less urgent or nonurgent)^15^; 4) consist of a diagnosis based on an International Statistical Classification of Diseases and Related Health Problems, 10th Revision, Canada (ICD-10-CA) from the list of Family Practice Sensitive Conditions; and 5) consist of a diagnosis code that is manageable by pharmacists in at least 1 Canadian jurisdiction, such as acne, eye conditions, skin conditions, cough, common cold and migraine.

For each ED visit, the patient’s postal code was used to determine the forward sortation area (FSA), which is a small geographic area that has about 15,000 persons and encompasses approximately 200 city blocks in urban areas.^16^ Scott’s Medical Database and the Health Workforce database were used to identify the number of physicians and nurse practitioners who practise in primary care in Ontario, including the FSA of their practice. Publicly available data from the Ontario College of Pharmacists’ website (www.ocpinfo.com) was used to identify licensed community pharmacies in Ontario and their postal codes. Aggregate-level community income was obtained from 2016 population census data at the FSA level. The median total household income at this level was determined, and communities were then categorized by quintile for use as the income indicator.

### Data analysis

All avoidable ED visits in Ontario were identified, and the rate of potentially pharmacist-manageable conditions among all avoidable visits was calculated. Then, the quintile of this rate was mapped to patients’ residential FSAs to describe the geographic distribution of pharmacist-manageable ED visits. Of the 523 FSAs in Ontario, 513 were included in the data provided by Statistics Canada, with 10 FSAs excluded because they were “not the dominant FSA in any dissemination area in the 2016 population census.”^17^

For each primary health care provider (family physicians and nurse practitioners), we extracted the FSA of their place of practice and then linked the number of all providers who practise in the same FSA to the population estimate and income quintile of that FSA. Similarly, for each community pharmacy, we extracted the FSA, then linked the total number of community pharmacies that are located in the same FSA. The mean distribution of family physicians and nurse practitioners working in primary care and the mean number of community pharmacies per 100,000 population was then calculated for each FSA rate quintile of potentially pharmacist-manageable conditions among all avoidable ED visits.

The differences in health care providers working in primary care and community pharmacies as well as income quintile distribution were determined using analysis of variance and chi-square, respectively, with a 0.05 level of significance. SAS version 9.4 (SAS Institute Inc, Cary, NC) was used to perform the statistical analysis, while ARCGIS version 10.7.1 (ESRI, Redlands, CA) was used to visualize the data (i.e., mapping).

### Ethics approval

This study was approved by University of Waterloo Office of Research Ethics (ORE file No. 22166).

## Results

**Figure 1:**
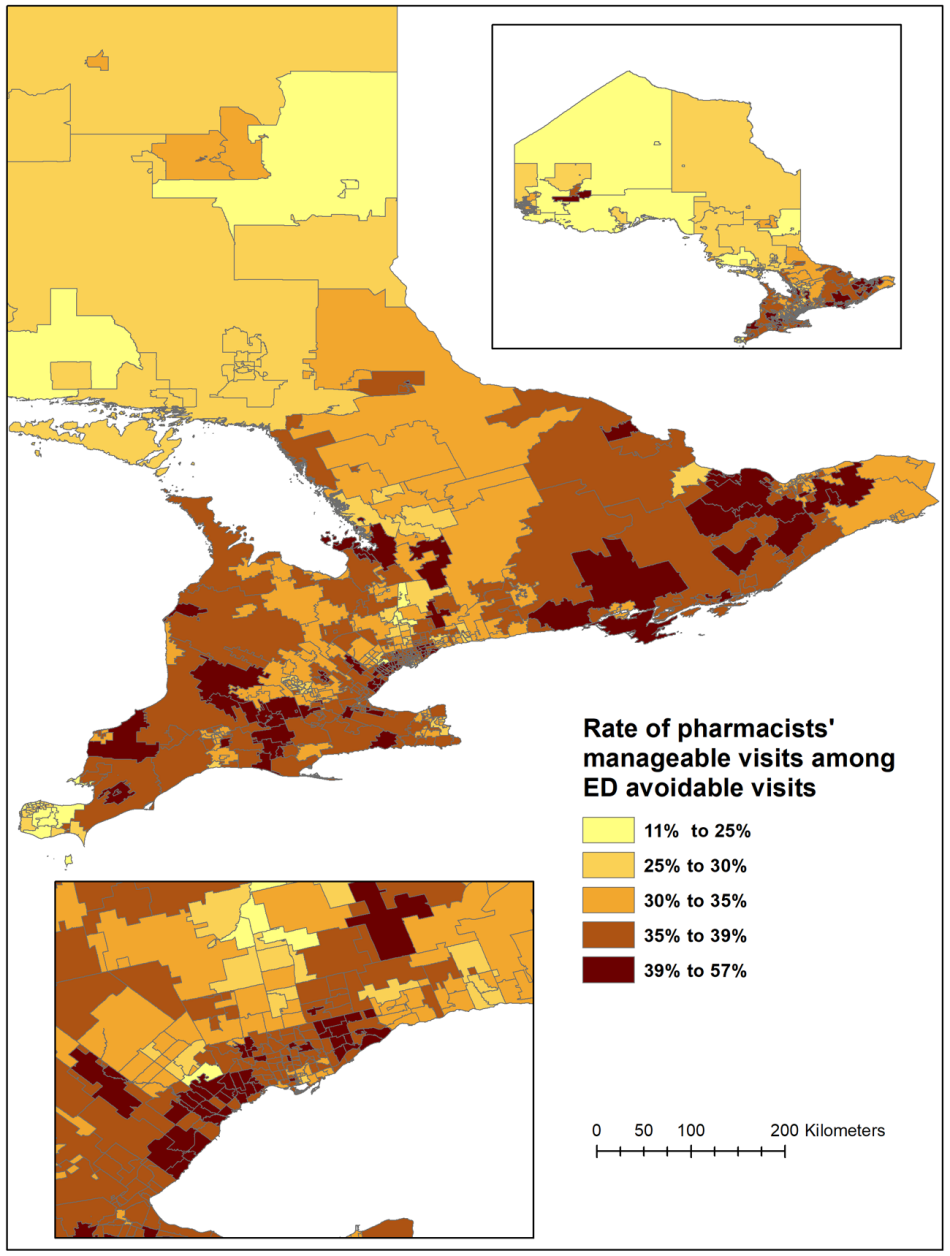
Spatial distribution of the rate of avoidable emergency department visits that are potentially manageable by pharmacists, by forward sortation area

During the study period, of a total of 34,550,020 ED visits in Ontario, 4,294,115 (12.4%) were considered avoidable. Of these,1,494,887 (34%) were deemed to be potentially pharmacist manageable and 1,449,324 (97%) were matched to FSA in the mapping analysis. Among all avoidable visits, the mean rate of potentially pharmacist-manageable visits was 35% (median 36%), and this rate ranged from 11% to 57% across Ontario FSAs (Figure 1).

Table 1 presents characteristics of FSAs (income, availability of physicians and nurse practitioners in primary care, and availability of community pharmacies) stratified across the 5 quintiles of the rate of potentially pharmacist-manageable ED visits. Here, it can be seen that there are significant differences in FSA income levels across the 5 quintiles of ED visits (*p* < 0.0001). Notably, FSAs with the lowest quintile of the rate of manageable visits also had more FSAs in the lowest quintile of income. Conversely, areas with the highest quintile of the rate of manageable visits had more FSAs in the highest quintile of income. No relationship was observed between potentially pharmacist-manageable ED visits and the availability of family doctors and nurse practitioners (*p* = 0.5940) or community pharmacies (*p* = 0.3018).

**Table 1:** Characteristics by forward sortation area (FSA), stratified by quintile of the rate of avoidable emergency department visits in Ontario that are potentially manageable by pharmacists

**Characteristic**	**Quintile of rate of all ED visits that could be managed by pharmacists**					**All FSAs (*n* = 513)**	***p* value**
	**Quintile 1 (lowest), *n* = 61 FSAs**	**Quintile 2, *n* = 103 FSAs**	**Quintile 3, *n* = 124 FSAs**	**Quintile 4, *n* = 136 FSAs**	**Quintile 5 (highest), *n* = 89 FSAs**		
Quintile of median total household income (*n*, %)							
Quintile 1 (lowest)	22 (36.1%)	30 (29.1%)	31 (25.0%)	28 (20.6%)	17 (19.1%)	128 (25.0%)	<0.0001
Quintile 2	12 (19.7%)	21 (20.4%)	32 (25.8%)	36 (26.5%)	13 (14.6%)	114 (22.2%)	
Quintile 3	9 (14.8%)	19 (18.4%)	21 (16.9%)	27 (19.9%)	23 (25.8%)	99 (19.3%)	
Quintile 4	9 (14.8%)	21 (20.4%)	23 (18.5%)	24 (17.6%)	18 (20.2%)	95 (18.5%)	
Quintile 5 (highest)	9 (14.8%)	12 (11.2%)	17 (13.7%)	21 (15.4%)	18 (20.2%)	77 (15.0%)	
Family physicians and nurse practitioners working in primary care per 100,000 population (mean, SD)							
	105 (105.6)	102 (97.3)	116 (105.7)	96 (101.2)	95 (88.7)	103 (100.0)	0.5940
Community pharmacies per 100,000 population (mean, SD)							
	40 (30.8)	37 (22.2)	38 (24.7)	33 (20.4)	35 (23.3)	36 (23.8)	0.3018

## Discussion

Depending on the geographic location, the proportion of avoidable ED visits by Ontarians during the study period that could have potentially been managed by a pharmacist under an ambulatory conditions program ranges from 11% to 57%. Across quintiles of the rate of potentially pharmacist-manageable ED visits, there were no differences related to the distribution of primary health care providers or the distribution of community pharmacies; however, a relationship was observed between household income quintile and ED visit quintiles. Here, lower quintiles of ED visits were associated with lower median household income, and higher quintiles of ED visits were associated with higher median household income.

We described the geographic distribution in the rate of pharmacist-manageable avoidable visits. The availability of other primary health care providers and community income level were not associated with areas of high rate of potentially pharmacist-manageable ED visits in Ontario. This is consistent with previous research that showed medical density (i.e., the distribution of physicians per 100,000 population) was not associated with inappropriate use of the ED.^18^ Although vulnerability, as measured by low income, was generally not associated with the rate of pharmacist-manageable visits, we found an overrepresentation of lower-income areas among FSAs with the lowest rate. Previous research found that low-income areas do not differ significantly in terms of access to primary care,^19,20^ despite having more unmet health needs.^21^ We found that despite low geographic accessibility to community pharmacies, low-income areas may have in fact a lower rate of potentially pharmacist-manageable visits.^11^ We were not able to assess other factors using our available administrative data.

We used data from a comprehensive database over 7 years to explore the spatial distribution of ED visits that can be potentially managed by pharmacists as part of an ambulatory conditions program. As such, our study is advantageous in enabling decision-makers to assess areas with the highest need of implementation of these services. However, several limitations should be noted. First, we identified potentially pharmacist-manageable ED visits using a list of conditions that can be managed by pharmacists in any Canadian jurisdiction outside of Ontario. However, the final list of conditions that will be pharmacist manageable in Ontario under the proposed ambulatory conditions/minor ailments legislation is still being finalized; therefore, our results likely represent an overestimation of actual benefits that may be observed under this program, because not all conditions within pharmacists’ scope of practice elsewhere are likely to be adopted here. In addition, there may be pharmacist-preventable conditions that we were not able to capture with the available data (e.g., adverse drug reactions, chronic disease complications or issues related to nonadherence), resulting in a potential underestimate of the rate of ED visits that can be affected by pharmacists. With regard to income categorization, we used the 2016 population census to assign areas’ income for 2010-2017, which may have caused misclassification of some areas’ income over the study period. However, it is not expected that areas will have significant changes in income level over 7 years, particularly relative to other areas. In addition, we used the average number of primary health care providers (family physicians and nurse practitioners) over the study period to determine availability, recognizing that changes in the health care workforce may have occurred over the study period, and we did not consider the geographic accessibility of these providers to the public.

## Conclusion

Among avoidable ED visits in Ontario, approximately one-third (range 11%-57%) are potentially pharmacist-manageble with an expansion to Ontario pharmacists’ scope of practice. Neighbourhoods with higher median household income were found to have higher rates of pharmacist-manageble conditions leading to an ED visit compared with lower-income neighbourhoods. Further research should examine systematic factors potentially contributing to the use of the ED for ambulatory conditions and the unique characteristics and perspectives of patients who access ED services for low-acuity conditions in “hot-spot” communities. ■
